# The double-corolla phenotype in the Hawaiian lobelioid genus *Clermontia* involves ectopic expression of *PISTILLATA* B-function MADS box gene homologs

**DOI:** 10.1186/2041-9139-3-26

**Published:** 2012-11-01

**Authors:** Katherine A Hofer, Raili Ruonala, Victor A Albert

**Affiliations:** 1 Department of Biological Sciences, University at Buffalo, Buffalo, NY, 14260, USA

**Keywords:** Myofascial pain, Trigger points, Neck/shoulder pain, Health status

## Abstract

**Abstract:**

**Background**

The Hawaiian endemic genus *Clermonti*a (Campanulaceae) includes 22 species, 15 of which, the double-corolla species, are characterized by an extra whorl of organs that appear to be true petals occupying what is normally the sepal whorl. Previous research has shown that the presence of homeotic petaloid organs in some other plant groups correlates with ectopic expression of B-function MADS box genes, but similar core eudicot examples of apparent groundplan divergence remain unstudied. B-function genes, which are not normally expressed in the sepal whorl, are required for determination and maintenance of petal identity. Here, we investigate the potential role of altered B-function gene expression contributing to the morphological diversity of this island genus.

**Results**

We examined the morphology and developmental genetics of two different species of *Clermontia*, one of which, *C. arborescens*, has normal sepals while the other, *C. parviflora*, has two whorls of petal-like organs. Scanning electron microscopy of cell surface morphologies of first and second whorl organs in the double-corolla species *C. parviflora* revealed conical epidermal cells on the adaxial surfaces of both first and second whorl petaloid organs, strongly suggesting a homeotic conversion in the former. Phylogenetic analysis of *Clermontia* species based on 5S ribosomal DNA non-transcribed spacer sequences indicated a probable single and geologically recent origin of the double-corolla trait within the genus, with numerous potential reversals to the standard sepal-petal format. Quantitative polymerase chain reaction analysis of homologs of the B-function genes *PISTILLATA* (*PI*), *APETALA3* and *TOMATO MADS 6* indicated ectopic expression of two *PI* paralogs in the first whorl of *C. parviflora*; no such homeotic expression was observed for the other two genes, nor for several other MADS box genes involved in various floral and non-floral functions. In the standard sepal-petal species *C. arborescens*, ectopic expression of PI homologs was not observed. In *C. parviflora*, the upregulation of *PI* homologs was precisely restricted to the perianth and stamen whorls, excluding a simple overexpression phenotype. *In situ* hybridization analysis of *C. parviflora* material similarly showed first and second whorl PI homolog expression in developing flower buds.

**Conclusions**

Our morphological and gene expression data strongly suggest that a drastic and heritable phenotypic change, at the level of floral groundplan, can originate from a homeotic mutation that is likely regulatory, being under precise spatiotemporal control as opposed to having pleiotropic characteristics. The uniqueness of this trait among core eudicots could be linked to increased ecological viability in an unstable island environment, a chance event which need not have posed any immediate adaptive benefit. We argue that the evolutionarily young morphological radiation of *Clermontia* may form a model system for general understanding of mechanisms of larger-scale angiosperm diversification in past, similarly unstable environments, in which small regulatory changes may have been responsible for modern-day groundplan differences.

**Trial registration:**

Clinical Trials.gov- NCT01710735

**Significance and Innovations:**

The present investigation is one of the first to examine the hypothesis of gross muscle contractile inhibition due to the presence of diagnostically relevant MFTrPs. Individuals suffering from clinically relevant levels of self-reported pain are able to tolerate maximum voluntary contraction testing, but delayed onset muscle soreness (DOMS) is a likely side-effect irrespective of symptom status. As a consequence, its confounding effect during subsequent testing must be taken into account.

## Background

The ABC model of floral development, which describes the role of three classes of organ identity genes in the spatial arrangement of floral organs, was proposed in accordance with studies in two model core eudicots, *Arabidopsis thaliana* and *Antirrhinum majus*[1,2]. In the determination of reproductive organs, expression of C-function genes alone specifies carpels and expression of both C-function and B-function genes gives rise to stamens. In the outer perianth whorl, sepal identity is determined by the lack of B-function and C-function genes. In the inner perianth whorl, the lack of C-function genes and the activity of B-function genes are required for determination and maintenance of petal identity [3]. All previously described B-function genes belong to the family of MADS-box containing transcription factors.

While the ABC model is well known, little is known about the evolutionary events that have led to the striking structural diversity among petaloid organs, including sepal or petal loss and transfer of petaloid characters between floral whorls. The presence of homeotic petaloid organs in other plant groups, such as the Ranunculales, monocots, magnolids and basal angiosperms, have been shown to be correlated with ectopic expression of B-function MADS box genes [4-7]. It has been suggested that similar core eudicot examples of apparent divergence from the standard condition, in which petaloid organs are found only in the second floral whorl, may be associated with heterotopic expression of B-function genes. However, these systems have remained inadequately studied.

Within the core eudicots, perianth organization rarely deviates from the standard sepal-petal format. In cases where ectopic petaloid organs do occur in core eudicots, the organs remain morphologically differentiated from petals, as compared with the identical perianth whorls that can often be observed in the monocots. Furthermore, petaloid organs which occur outside of the inner perianth whorl, such as petaloid bracts in dogwood (*Cornus*), the petaloid sepal in *Impatiens*, and sepal-derived and stamen-derived petaloid organs in the Caryophyllales, have revealed employment of alternate petal identity programs in the formation of petaloid organs, discounting simple extension of B-class gene expression in these systems [8-11]. Similarly, some monocots have been shown to lack expression of B-function genes in the outer tepals [12].

**Figure 1:**
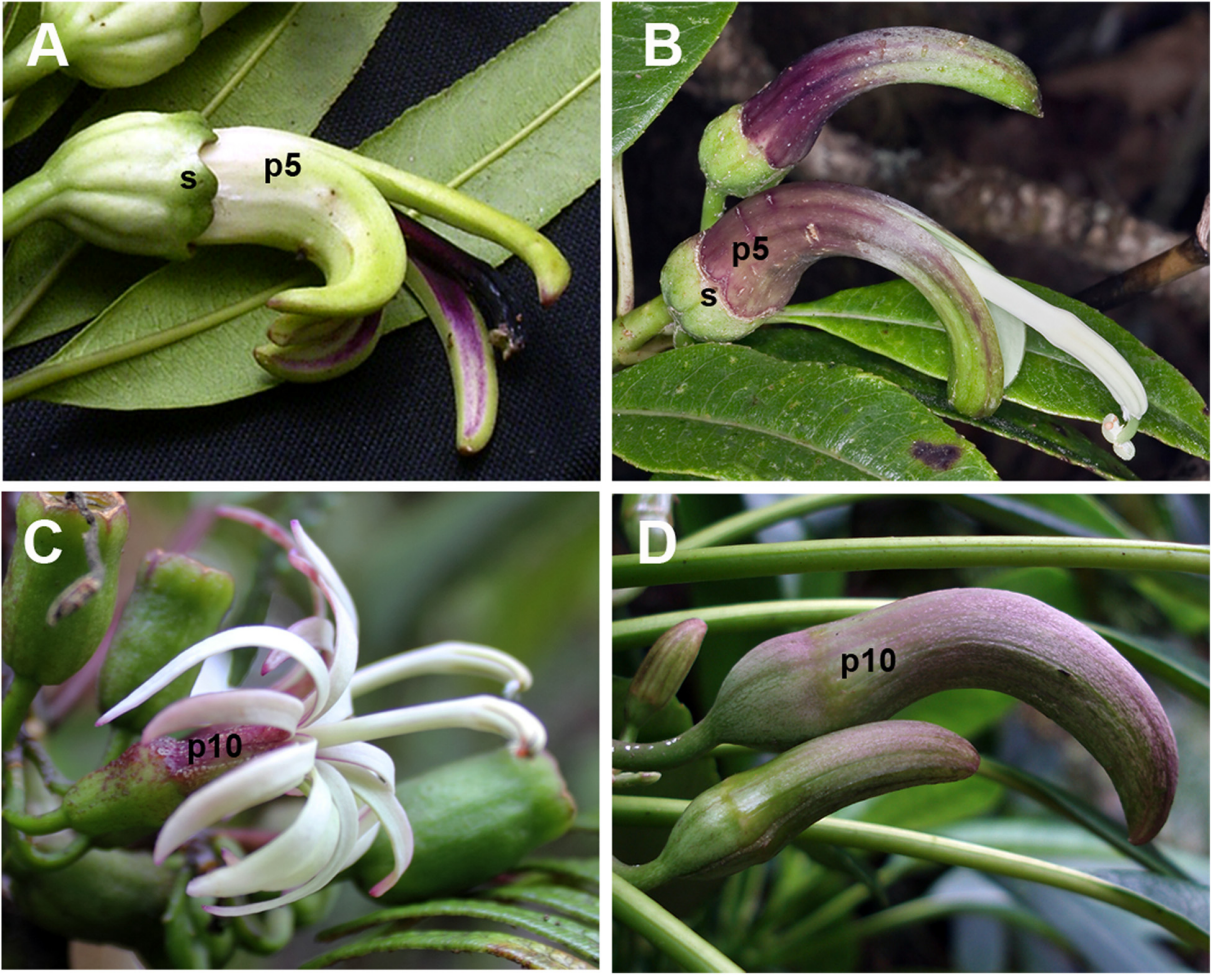
**Groundplan diversity in representative *Clermontia *species.** Floral morphology of the standard sepal-petal species (**A**) *Clermontia arborescens* and (**B**) *C. fauriei* and double-corolla species (**C**) *C. parviflora* and (**D**) *C. oblongifolia* are shown. *C. arborescens* and *C. fauriei* exhibit small green sepals (s) in the outer perianth whorl and five large showy petals (p5) in the inner perianth whorl. *C. parviflora* and *C. oblongifolia* bear 10 petal-like organs (p10) in both perianth whorls. Photos courtesy of Anne-Cathrine Scheen (**A**), John Game (**B**) and Jupiter Nielsen (**C**,**D**).

The Hawaiian endemic lobelioids, Campanulaceae, comprise 6 genera and 126 species and represents the largest plant radiation on any ocean island archipelago. *Clermontia*, a genus within the lobelioids, includes 22 species of bird-pollinated trees and shrubs possessing morphologically diverse flowers [13]. *Clermontia* flowers exhibit inferior ovaries and produce fleshly fruits. A drastic homeotic phenotype, in which sepals are replaced by an extra whorl of organs that appear to be true petals, characterizes 15 of the 22 species: the double-corolla species. This petal-petal format is a unique trait within the standard groundplan, consisting of a sepal-petal perianth, of the core eudicots. For most double-corolla *Clermontia* species, the sepal-to-petal transformation is a complete one-for-one conversion. *C. arborescens* demonstrates the ancestral condition, displaying an outer perianth whorl of small green sepals and larger, showier petal organs in the inner whorl (Figure 1A). The identical first and second whorl organs present in *C. parviflora*, both of which are petaloid, reveal the double-corolla phenotype (Figure 1C). Two other *Clermontia* species are also figured to make the point about the double-corolla phenotype: *C. fauriei* (Figure 1B), with tiny sepals and one petal whorl, is otherwise very similar to *C. oblongifolia* (Figure 1D), which bears only petal-like organs.

The genus *Clermontia* is endemic to the Hawaiian Islands, which allows for a system that can be studied on a recent and established evolutionary timescale. The Hawaiian Islands were formed by movement of the Pacific plate over a mantle plume; this resulted in islands that emerged from volcanic eruptions and erode in a southeast to northwest geographic progression. Further, unstable environments may allow for increased ecological viability of drastic groundplan differences that may not necessarily create any immediate increase in fitness. This may account for the success of a morphological radiation where a drastic groundplan difference is caused by small regulatory changes. The naturally occurring homeotic mutation in *Clermontia* offers an appropriate model for studying the potential role of altered B-function gene expression contributing to heterotopic petaloid organs within the core eudicots [14], and provides an example that may have general implications for the study of island radiations.

## Methods

### Scanning electron microscopy

RNAlater-preserved samples (Applied Biosystems, Carlsbad, CA, USA) were dissected to separate inner and outer perianth organs. Tissues were dehydrated in a graded ethanol series and chemically dried with hexamethyldisilazane as described in Costea *et al*. [15]. Samples were mounted on stubs, sputter coated with evaporated carbon, and micrographs were taken using a Hitachi-S-4000 scanning electron microscope (Hitachi, Ltd., Tokyo, Japan). Both adaxial and abaxial surfaces were analyzed.

### Sequence generation and phylogenetic analysis

Genomic DNA was extracted from herbarium- or silica-preserved vegetative material using Qiagen DNeasy Plant Kits (Qiagen, Valencia, CA, USA). Isolation was performed with the use of universal 5S ribosomal DNA non-transcribed spacer (5S-NTS) primers previously described in Cox *et al*. [16]. Amplicons were cloned into the PDrive cloning vector (Qiagen) and five to eight colonies per sample were sequenced. 5S-NTS sequences were submitted to GenBank and have accession numbers [GenBank:JQ734775] to [GenBank:JQ734917]. B-function MADS box genes that were not obtained from 454 transcriptome sequencing (KAH and VAA, unpublished results) were isolated using RT-PCR with previously reported primers [17]. To gain insight into the relationships among the 5S-NTS sequences, a maximum likelihood phylogenetic tree was constructed. Similarly, a maximum likelihood tree was generated comprising the two *Clermontia PI* homolog duplicates as well as *PI* homologs from selected other taxa, including Campanulaceae, Asteraceae and outgroups. Sequences were aligned using MUSCLE with default settings [18]. The sequence alignments, with complete taxon names, voucher information (where available) and GenBank accession numbers are available in Additional files 1 and 2. A single most optimal tree for each data set was computed using the RaxML BlackBox web server (http://phylobench.vital-it.ch/raxml-bb/) running RaxML version 7.2.8 [19]. Default settings were used with the general time reversible plus gamma model of molecular evolution. One hundred bootstrap samples were generated to assess support for the inferred relationships. Local bootstrap values (in percentages) are indicated for branches with >50% support.

### Quantitative polymerase chain reaction

RNAlater-preserved tissues (except for young leaves, these were floral organs from buds in various pre-anthesis stages) were dissected into organ groups as needed, total RNA was isolated using a Qiagen RNeasy Plant Kit, and mRNA was subsequently isolated using a Qiagen Oligotex mRNA Kit. Reverse transcription of mRNA with a Bio-Rad iScript cDNA Synthesis Kit (Bio-Rad, Hercules, CA, USA) was performed to synthesize cDNA. Each reaction comprised 20 ng of cDNA. Primers were designed from sequences of B-function and other MADS box genes discovered either by transcriptome sequencing (KAH and VAA, unpublished data) or isolated by RT-PCR and are listed in Additional file 3. Primers for each *PI*-like homolog yielded unique sequences when checked for specificity. Amplification and real-time detection was performed using Bio-Rad iQ SYBR Green Supermix on an MyiQ2 Real-Time Detection Thermal Cycler (Bio-Rad). Standardization was performed using β-actin to determine delta-delta critical threshold values. Bars represent standard deviations based on means of three independent experiments, each of which included two replicates. Gene expression is presented in as fold differences relative to expression in the highest expressing organ in each gene category, which was set to 10.

### *In situ* hybridization

*Clermontia* flower buds were fixed with formalin-acetic acid-alcohol (FAA), embedded in paraffin and sectioned using standard methods. *In situ* hybridizations were performed essentially as described in Ruonala *et al*. [20]. For the post-hybridization washes, an InsituPro VsiVSi 3.0 (Intavis AG, Koeln, Germany) device was used with the protocol shown in Additional file 4. The color reaction to visualize hybridized probe was done for approximately 24 hours at room temperature. Sections were photographed using an AxioImagerZ1 (Zeiss, Munich, Germany) equipped with AxioCam MRc (Zeiss). The digoxigenin-labeled probe covered 430 bp of the *Clermontia PI* coding region, excluding the MADS domain. Primers used to amplify the *Clermontia PI* probe fragment were: forward, 5^′^ GCATGAGTACTGTAGCCCTTCC 3^′^; reverse 5^′^ GAAGAGTGGGGAAGGAAGATTT 3^′^.

## Results

### Perianth epidermal morphology

**Figure 2:**
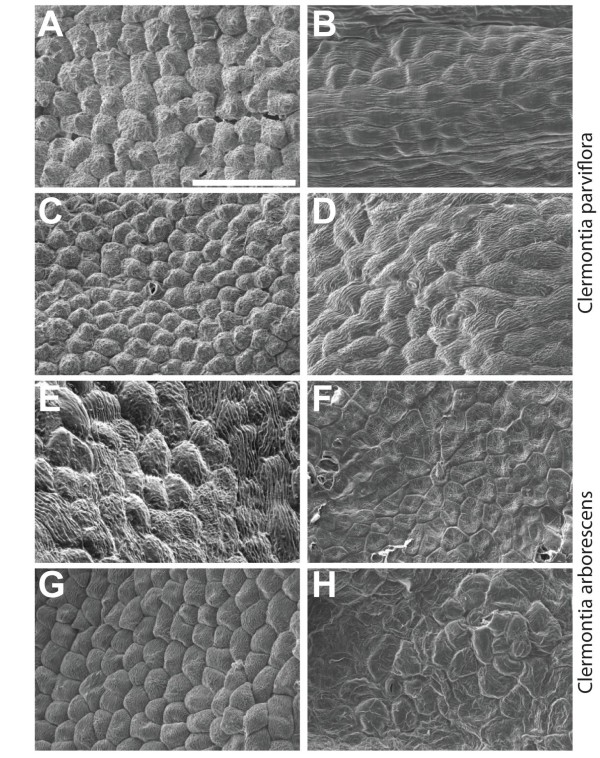
**Epidermal micromorphology of perianth organs.** Scanning electron microscope images from (**A**-**D**) *C. parviflora* and (**E**-**H**) *C. arborescens* on the adaxial (left panels) and abaxial (right panels) epidermal surfaces. Canonical Conical epidermal cell morphology, an indicator of petal identity, is discovered are apparent on the adaxial surface of the outer perianth organs of *C. parviflora* (**A**). Similar cone-shaped cells are observed on the adaxial surface of inner perianth organs in both *C. parviflora* (**C**) and *C. arborescens* (**G**). However, the outer perianth organs of *C. arborescens* display a flattened morphology more typical of sepal organs (**E**). Scale bar denotes 100 μm.

Scanning electron microscopy experiments were performed on the adaxial and abaxial surfaces of organs located in the outer two floral whorls for standard sepal-petal and double-corolla flowers. Cell surface morphologies of first and second whorl organs of the double-corolla species *Clermontia parviflora* revealed conical epidermal cells on the adaxial surfaces of both first and second whorl petaloid organs, strongly suggesting a homeotic conversion in the former (Figure 2A,C). While the inner whorl of the standard bipartite perianth species, *C. arborescens*, revealed conical epidermal cells on the adaxial surface, the outer whorl epidermal cells lacked the well-defined conical shape; such flattened, irregular cell morphologies are typical for sepal organs (Figure 2E,G).

### Phylogenetic analysis

**Figure 3:**
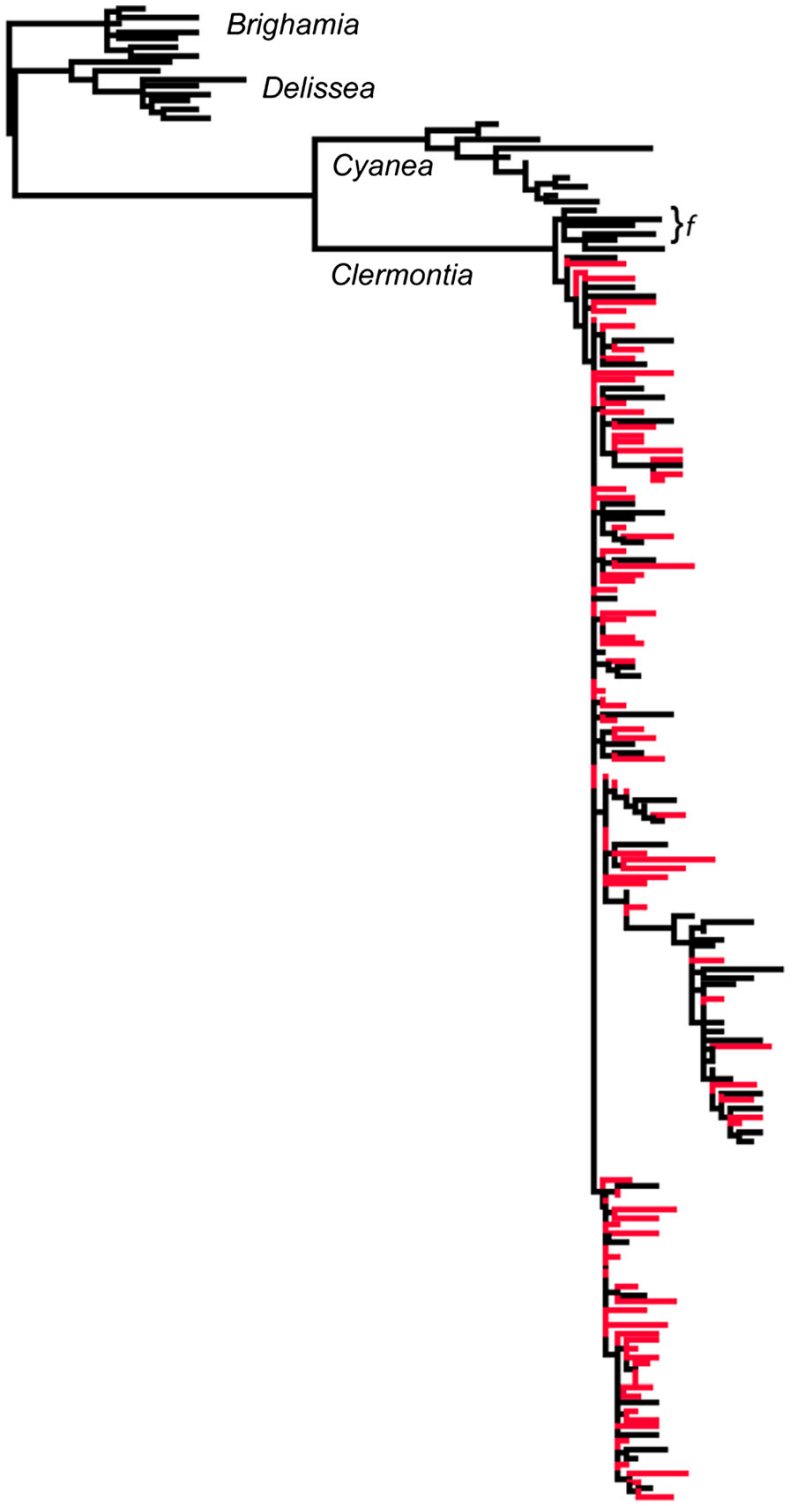
**Schematic representation of phylogenetic analysis of 5S**-**NTS ribosomal DNA sequences.** Outgroups *Brighamia* and *Delissea* are displayed along with *Clermontia*’s sister genus *Cyanea*. *C. fauriei* (f) exhibits the standard sepal-petal groundplan and forms a distinct group separate from all other *Clermontia* species, including both sepal-petal and double-corolla species. Red indicates sequences derived from double-corolla DNA samples.

Phylogenetic analysis of 5S-NTS sequences shows clear divergence of *Clermontia* from outgroup genera *Brighamia* and *Delissea*, and *Clermontia*’s sister genus *Cyanea*, and demonstrates extremely low sequence divergence among *Clermontia* species (Figure 3). The single-corolla species *C. fauriei*, the only species found on the oldest island, Kauai, forms a significantly distinct group from other *Clermontia*, which include both single- and double-corolla species (Figure 3 and Additional file 5). This indicates a probable single and geologically recent origin of the double-corolla trait within the genus, likely by the time of the emergence of Oahu (*circa* 3.5 million years ago), with numerous potential reversals to the standard sepal-petal format. Oahu holds two species with the double-corolla phenotype, *C. persicifolia* and *C. oblongifolia*, the former of which is endemic to that island. 5S-NTS sequence details and an alignment are presented in Additional files 1 and 6.

### Expression analysis

**Figure 4:**
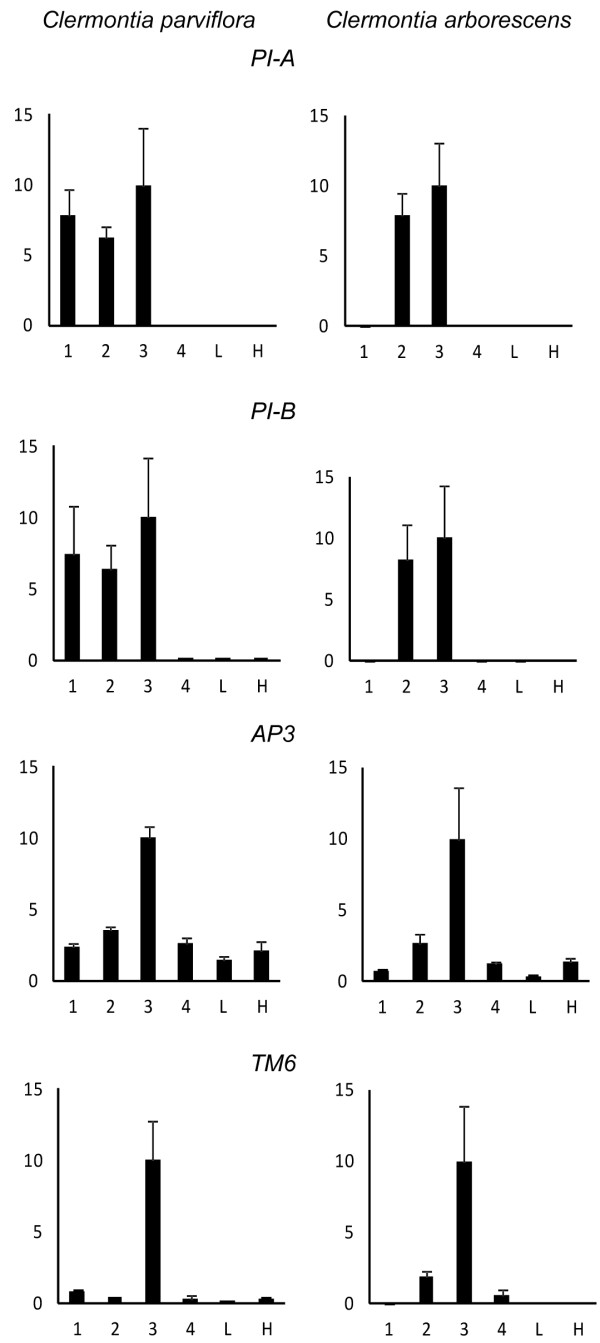
**Quantitative gene expression of B**-**class genes in dissected floral organs.** Expression of both *PISTILLATA* (*PI*) homologs is constrained to whorls 2 and 3 of *Clermontia arborescens* (top two right panels) but is extended to whorls 1, 2 and 3 in *C. parviflora* (top two left panels). No substantial difference in expression pattern is observed in other B-function genes, *AP3* and *TM6* homologs (bottom two panels). β-actin was used as an internal control. Bars indicate mean and standard deviation for three independent experiments each including two replicates. 1, outer perianth; 2, inner perianth; 3, stamen; 4, carpel; L, leaf; H, hypanthium.

qPCR analysis performed on homologs of the B-function genes *PISTILLATA* (*PI*), *APETALA3* (*AP3*), and *TOMATO MADS 6* (*TM6*) was used to elucidate expression patterns in outer perianth, inner perianth, stamen, carpal, vegetative apices and hypanthium (inferior ovary) tissues of the double-corolla species *Clermontia parviflora* and the standard sepal-petal species *C. arborescens*. This analysis indicated ectopic expression of the two *PI* paralogs in the first whorl of *C. parviflora*; no such drastic homeotic expression was observed for the other two genes, although *AP3* expression appears to be slightly elevated relative to *C. arborescens* (Figure 4). Transcriptional analysis of other *Clermontia* MADS box gene homologs, *SHORT VEGETATIVE PHASE* (*SVP*), *SUPPRESSOR OF OVEREXPRESSION OFCONSTANS* (*SOC1*), *AGAMOUS-LIKE 6* (*AGL6*) and *SEPALLATA3* (*SEP3*), which are involved in various floral and non-floral functions [21-24], was also performed. No remarkable differences were found between expression patterns in these genes between sepal-petal and double-corolla floral organs (Additional file 7). In *C. arborescens*, detection of *PI* transcripts was restricted to the inner perianth organs and stamens, with no ectopic expression of *PI* homologs being observed in whorl 1, which is consistent with eudicot-standard lack of B-function genes in sepals (Figure 4). No expression was detected for either of the *PI* homologs in the carpel organs of *C. parviflora* (Figure 4), indicating that the up- versus downregulation of the *PI* homologs is precisely constrained to the perianth and stamen whorls, excluding a simple overexpression phenotype that would be expected to engender a pleiotropic phenotype.

**Figure 5:**
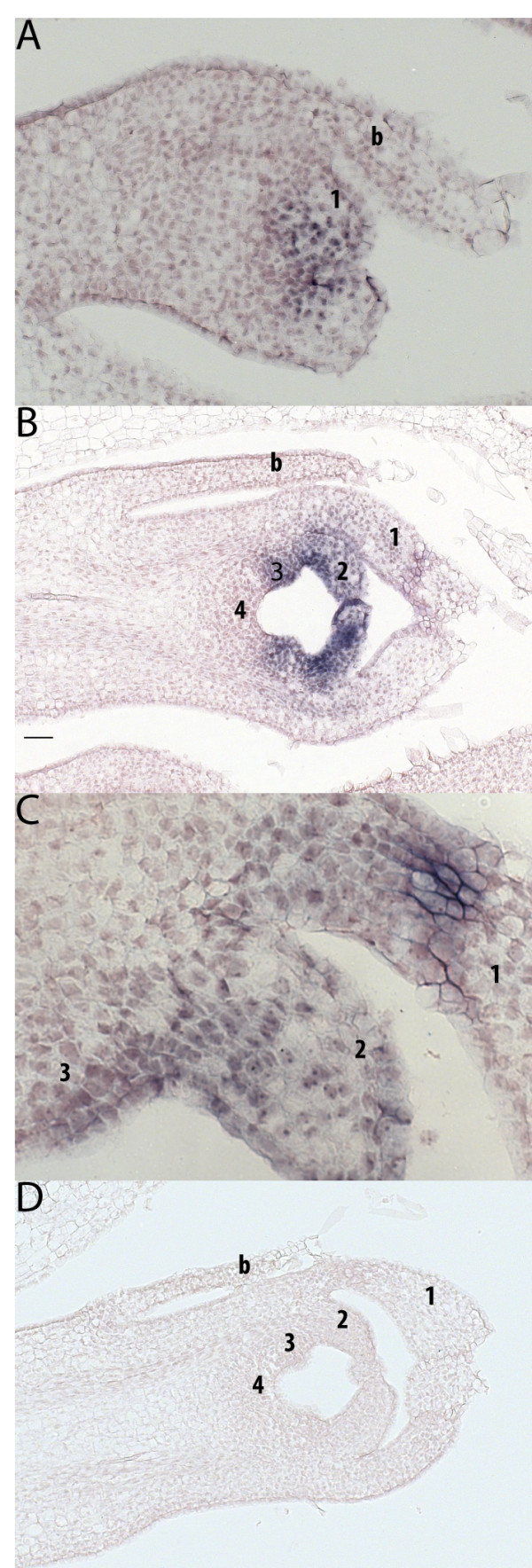
***In situ *analysis of *PISTILLATA *expression in flower primordia of *Clermontia parviflora. **** In situ* hybridization on flower primordia of *C. parviflora* displays expression of *PI* homologs in (**A**) early stage outer perianth organs and (**B** and **C**) in both perianth organs and whorl 3 in a late-stage bud. (**D**) Sense *PI* homolog controls show low background staining. Images are displayed at 20× (A), 10× (**B** and **D**) and 40× (**C**). Organs are numbered by whorl; b = bract.

*In situ* hybridization was used to study expression of both *PI* homologs in primordial stages of floral organ development of the double-corolla species *Clermontia parviflora*. Consistent with qPCR data, this analysis showed ectopic expression of *PI* homologs in the outer perianth whorl of developing double-corolla flower buds, as well as in the petal and stamen whorls, as expected (Figure 5).

## Discussion

The petals of angiosperm flowers, laminar organs occupying the second floral whorl, often share characteristics such as expression of B-function MADS genes, showy morphology, pigmentation and production of aromatic compounds. However, petals exhibit substantial diversity and organs with petaloid characteristics can be found outside of the second floral whorl, making the definition and identification of these organs rather challenging. The adaxial epidermal surfaces of petals are frequently composed of conical cells whereas those of other floral and vegetative cells are typically not [25]. Identification of petaloid organs has been determined by observation of conical epidermal cells in cases of homeotic conversions involving petals [26,27]. Analysis of micromorphological characteristics on the adaxial epidermal surfaces of first-whorl organs in the double-corolla species *Clermontia parviflora* reveals conical cells, a marker of petal identity. This supports the hypothesis of homeotic transformation of sepals to petals.

Based on the phylogenetic analysis presented here and the geographic distribution of *Clermontia*, the origin of this homeotic transformation was likely established via a single and geologically recent occurrence. The Hawaiian Islands were formed by the northwestward movement of the Pacific tectonic plate over a fixed volcanic plume, resulting in a layout where the islands progress from oldest to youngest in a northwest to southeast manner [28]. *C. fauriei* is the only species found on Kauai, the oldest of the Hawaii Islands, and it displays the ancestral sepal-petal format. The neighboring island Oahu is home to five species, including *C. fauriei* and four double-corolla species [29]. 5S-NTS data support previous findings suggesting *Clermontia*’s substantial sequence divergence from its sister genus *Cyanea* but low sequence divergence within the genus [30]. Our data do indicate sequence divergence between *C. fauriei* and all remaining *Clermontia* species, including both standard sepal-petal and double-corolla species, perhaps reflective of a split age at least by the time of the origin of Kauai. The phylogenetic clustering of ancestral state and petal-petal format species observed suggests multiple potential reversals occurred during the radiation of *Clermontia*. Similar findings on Hawaiian lobelioid inter-relationships, including evolution of reversals in the *Clermontia* clade, have been reported by Givnish and collaborators [13,31,32].

The transformation of first-whorl sepal organs into organs bearing petal identity in double-corolla *Clermontia* species, which is often complete, led us to question whether this phenomenon may be regulated by ectopic expression of B-class genes. Our detection of ectopic expression of *PI* homologs in floral primordia indicates a likely role of *PI* in the differentiation and determination of outer whorl floral organs, resulting in the development of petal identity. *Lacandonia schismatica* (Triuridaceae) exhibits a homeotic transformation in which central stamens are surrounded by carpels. The simple displacement of the B-function has been shown to play a role in this morphological shift [33]. In dove tree (*Davidia*), early B-class gene expression in petaloid bracts has been suggested to lead to a partial petaloid phenotype; however, the lack of late-stage expression makes the mechanism unclear [34]. In *Clermontia*, not only is expression of *PI* homologs detected in floral primordia, but we have also shown continued expression in late-stage outer whorl floral organs, supporting the function of B-class genes in the maintenance of homeotic petal identity. Furthermore, we show that expression of a variety of other MADS-box gene homologs involved in various floral and non-floral functions, specifically *AP3*, *TM6*, *SEP3*, *AGL6*, *SVP* and *SOC1*, present expression patterns that are largely or completely consistent between standard sepal-petal and double-corolla species (Additional file 7).

Expression of a *Clermontia AP3* homolog is detected in the outer whorl of both standard groundplan species and double-corolla species. Therefore, we hypothesize that ectopic expression of *PI* homologs would be sufficient to induce the obligate AP3-PI heterodimer autoregulatory feedback loop. In *Arabidopsis*, expression of *PI* alone is not able to induce petaloidy in vegetative organs; however, it is when co-expressed with *AP3* in vegetative organs or expressed alone in outer whorl perianth [3]. The expression of an *AP3* homolog in the outer whorl perianth in *Clermontia* indicates that the genetic background condition required for ectopic *PI* expression to induce petaloidy is present. *SEP3* is not able to induce petaloidy on its own, but has been shown to increase petaloid characteristics of ectopic petaloid organs, and the heterodimer AP3-PI forms a ternary complex with SEP3 [35,36]. We hypothesize that in *Clermontia*, low-level expression of *SEP3* and *AP3* homologs in the ancestral condition would set up a context in which ectopic expression of *PI* would be enough to induce petal identity in the outer whorl perianth.

Gene duplications among B-class genes, resulting in the euAP3, TM6 and PI groups, have been shown to be of evolutionary significance in the production of floral diversity. It has been suggested that extension of the B-class gene model has played a diversity-generating role in cases with a history of gene duplication among *PI* homologs or *AP3* homologs in taxonomic groups with otherwise undifferentiated first and second whorls [37]. Here, we demonstrate ectopic and sustained expression of both *Clermontia PI* homolog duplicates, which clearly derive from a lineage-specific duplication within Campanulaceae (Additional file 8). This apparent subfunctionalization event following gene duplication may have played a key role in the events following *PI* duplication in *Clermontia*. The two homologs may be largely functionally redundant, or one may be expressed first and induce the expression of the other, consistent with an autoregulatory feedback loop when obligatory dimerization with AP3 occurs.

The precise regulation that restricts ectopic expression of *PI* homologs to the first-whorl primordia in early and late-stage organs, as seen in *Clermontia parviflora*, indicates that small regulatory changes may be responsible for the dramatic change in the floral groundplan established in eudicots. This tight spatiotemporal regulation allows for the deployment of a drastic homeotic mutation without causing potentially deleterious pleiotropic effects, such as transformation of the carpel whorl into stamens. In *Arabidopsis*, ectopic expression of B-function genes has been shown to be sufficient to transform the carpel whorl into staminoid organs [3]. The specific regulation of this heritable mutation is necessary to produce viable organisms, since simple overexpression would be likely to cause infertility through the production of staminoid carpels. Other viable homeotic transformations within the angiosperm groundplan have occurred but have not led to radiations as seen in *Clermontia*.

The double-corolla phenotype may not have initially acted with adaptive advantage, its success perhaps relying instead on passive expansion of the mutation by random genetic drift in small populations in the unstable environments of the Hawaiian Islands. The sepal-petal ancestral status of *Clermontia* is supported by our phylogenetic analysis and the uniformly eudicot-standard floral morphology of its sister genus *Cyanea* and further closely related outgroup lobelioid species. While phylogenetic analysis demonstrates multiple possible reversals to the ancestral condition, the radiation of the double-corolla mutation indicates the long-term viability of the mutation. This gives insight into possible mechanisms that may have shaped diversity among floral groundplans in past unstable environments; small regulatory changes, as opposed to large coding-sequence differences, could account for morphological differentiation in other island groups such as the Hawaiian silverswords, Hawaiian mints and Canarian Crassulaceae.

## Conclusions

Our morphological and gene expression data strongly suggest that a drastic and heritable phenotypic change, at the level of the floral groundplan, can originate from a homeotic mutation that is likely regulatory, being under precise spatiotemporal control as opposed to having pleiotropic characteristics. The uniqueness of this trait among core eudicots could be linked to increased ecological viability in an unstable island environment, a chance event that need not have posed any immediate adaptive benefit. We argue that the evolutionarily young morphological radiation of *Clermontia* may form a model system for general understanding of mechanisms of larger-scale angiosperm diversification in past, similarly unstable environments, in which small regulatory changes may have been responsible for modern-day groundplan differences.

## Abbreviations

Bp: base pairs; qPCR: quantitative PCR; RT-PCR: reverse-transcriptase polymerase chain reaction; 5S-NTS: 5S ribosomal DNA non-transcribed spacer.

## Competing interests

The authors declare that they have no competing interests.

## Authors' contributions

VAA designed and led the study; RR performed *in situ* hybridization experiments; KAH executed all other experiments, analyzed data and drafted the manuscript. All authors read and approved the final version.

## Supplementary Material

Additional file 1 **5S-NTS rDNA sequence alignment.** Accession numbers and sequence data, as a MUSCLE alignment, are displayed in FASTA format. Click here for file

Additional file 2 **Coding-sequence alignment of *PISTILLATA*-like genes from *Clermontia *and outgroups.** Accession numbers and sequence data, as a MUSCLE alignment, are displayed in FASTA format. Click here for file

Additional file 3 **Primers used for quantitative RT-PCR.** Amplification of target and control genes was performed using the primer sequences listed in table format. Click here for file

Additional file 4 **Command file for *in situ *hybridization.** The method for *in situ* hybridization using the InsituPro Vsi 3.0 (Intavis AG) liquid handling robot is presented. Click here for file

Additional file 5 **Phylogenetic analysis of 5S-NTS rDNA sequences.** The single tree of maximum likelihood is shown with branches supported by bootstrap values >50% so indicated. Click here for file

Additional file 6 **5S-NTS rDNA sequence data.** Accession numbers and sequence data are displayed in table format. Click here for file

Additional file 7 **Quantitative RT-PCR for MADS-box containing genes.** Expression patterns of *SVP*, *SOC1*, *AGL6*, and *SEP3* homologs in floral whorls 1,2,3,4, leaf and hypanthium for sepal-petal *C. arborescens* and double-corolla *C. parviflora* show no significant differences between species. Expression levels are shown as fold differences relative to β-actin and represent the mean and standard deviation of three independent experiments with two replicates each. Click here for file

Additional file 8 **Phylogenetic analysis of *PISTILLATA*-like coding sequences from *Clermontia *and outgroups.** Based on these data the two *Clermontia* duplicates clearly arose after common ancestry with *Campanula*, a close sister taxon in Campanulaceae, although it cannot be certain that additional copies remain undetected in this and other species, such as with *Nyssa* in Cornales. No other *PI*-like sequences from the same plant individuals represented here are available in GenBank. *Clermontia parviflora* PI-like copies are shown. The single tree of maximum likelihood is figured with bootstrap values indicated. Click here for file
