# Supplementary material for: The double-corolla phenotype in the Hawaiian lobelioid genus Clermontia involves ectopic expression of PISTILLATA B-function MADS box gene homologs
Source: EvoDevo. 2012 Nov 1;3:26. doi: 10.1186/2041-9139-3-26 (PMC3564722; doi:10.1186/2041-9139-3-26)
Supplement: Additional file 2 — Coding-sequence alignment of PISTILLATA-like genes from Clermontia and outgroups. Accession numbers and sequence data, as a MUSCLE alignment, are displayed in FASTA format. [file 2041-9139-3-26-S2.doc]

>Helianthus annuus AY173069

CACAGGCTAGTAACATACTCCAAAAGAAAGAATGGAATCATCAGAAAAGCTAAGGAGATTACTGTTCTCTGTGATGCTAATGTCTCTCTTGTTGTATATGGATCTTCTGGCAAGATGTATGAGTACTGCAGCCCCAATACCAACTTGACTGACATGCTGGATAGATATCCAAGGCTTTCTGGAAATAAGTTGTGGGATGCTAAACATGAGAATTTGCAGATTGAAATTGACAGAATCAAGAAAGAGAATGAGAGCATGCAAATTGAGCTCAGGCACTTGAAAGGGGAAGATATAACATCTTTGAACTATGAAGAACTAATTGCATTTGAAGATGCACTTGAAAATGGACTTACCCACATTCGTGAGAAAAAGGATGAAATCCCCCAAATTATGAGGAAGCATGAACAAGTTCTAGAGGAGGAGAATAAGCACCTAATGTATATGGTGCAACAAAGTGAAATGGCAGCAATGGGAGAT---------------------------------------------------TAC---CAAGCAGAT---------GGACCCTTTTCTTTCCGTGTCCAACCGATGCAGCCGAACTTGCATGAGAGGATG

>Gerbera hybrida AJ009726

AACAGGCAAGTCACCTACTCAAAGAGAAAGAATGGCATCATCAAGAAAGCTAAAGAAATTACTGTTCTTTGTGATGCTAATGTTTCCCTTGTTATCTATGGCTCTTCTGGCAAGATGTATGAGTATTGTAGCCCCAAAACCAATTTGATTGACATGCTGGATCGCTATCAAAGGCTTTCTGGAAACAAGTTGTGGGATGCTAAACATGAGAATCTCCAGAATGAAATTGATAGAATCAAGAAAGAGAACGAGAGCATGCAGATTGAGCTAAGGCACTTGAAAGGGGAAGATATAACATCTTTGAACTATGAAGAACTGATTGCCTACGAAGATGCCCTTGAAAATGGACTCACCAATATTCGTGAAAAGAAGGATGAAATCCCAAAAATCATGAGGAAACATGAGCAAGTTCTGGAGGAGGAAAATAAGCACCTTATGTATTTAGTGCAACAAAGTGAGATGGCAGCCATGGGAGAT---------------------------------------------------TAC---CAAGCAGCTCACGAG------CCTTTCTCCTTCCGTGTCCAGCCAATGCAGCCTAATTTGCATGAGAGGATG

>Campanula latifolia HQ853407

------------------------------AATGGGATCATCAAGAAAGCTAAAGAAATCACCATTCTTTGTGATGCTAAGGTCTCCTGTGTCATCTATGGTGGTTCTGGCAAAATTTATGAGTATTGTAGCCCTTCTACCAACTTAGCTGACATGTTGGACAAATACCAAACTCTTTCTGGGAAGAAGCTCTGGGATCCTAAGCATGAGAATCTGAGCAATGAAATTGAGAGAGTGAGGAAGGAGAATGAAAATATGCAGATTGAGCTCAGGCACTTGAAGGGAGAAGACATCACATCTTTGACTCACAAAGAACTTCAAGCATTTGAAGACGCTCTTGAAAATGGCCTTACAAGCATTCGTGCCAAAAAGTCTGAGATCCCAAAGATGATGAGCAAACATGGCCAAATACTGGAGGAGGAGAATAGGCATCTCCAGTATATTCTGCAACAAAATGAGATGGCAGCTATGGAAGGAAACATGGCG---CAAATGGAGATGAACGGATATCATCAGAGAATG---------AATCAGGCCGATCATGATCAGCTGCCCTTCTCATTCCGTGTTCAACCCATTCAGCCGAATTTACACGAGCGCATT

>Clermontia parviflora A JX519567

AATCGGCAGGTGACGTATTCGAAGCGGCGAAATGGGATCATCAAGAAAGCTAAAGAAATCACAATTCTTTGTGATGCCAAGGTCTCTCTTGTTATCTATGGAACTTCTGGCAAGATGCATGAGTACTGTAGCCCTTCCACTAATCTAGTTGACATGCTGGATCGATACCAAACTCTTTCTGGGAAGAAGCTCTGGGATGCTAAGCATGAGAATCTCAACAATGAAATTGCGAGAATCAAGAAGGAGAATGACAATATGCAGATTGAACTCAGGCACTTGAAGGGAGAAGATATAACATCTTTGCATCACAAAGACCTAATCGCTCTTGAAGATGCGCTTGAGAACGGACTTTTGAACATACGTAGCAAAAAGTCCGAGATCCCAAAGATGATGAGCAAACATGGACAAATATTAGAGGAGGAGAACAGGCACCTCCAGTATATAGTGCAACAAACTGAGATGGCAGCTATGGAAGGTAACGTGGCAGATCAGATGGAC---AACGGGTACCATCAGAGAATGAGAGACTACAACCAACCAGATCAT---CAGGTGCCCTTTGCGTTTCGCGTTCAGCCAATTCAGCCAAATCTACACGAGCAGATC

>Clermontia parviflora B JX519568

AACAGGCAAGTGACGTACTCGAAGAGGCGAAATGGGATCATCAAGAAAGCTAAAGAAATCACTATTCTTTGTGATGCCAAGGTCTCTCTTGTTATCTATGGCACTTCTGGCAAGATGCATGAGTACTGTAGCCCTTCCACTAATTTAGTTGACATGCTGGATCGATACCAAACTCTTTCTGGGAAGAAGCTCTGGGATGCTAAGCATGAGAATCTCAATGGTGAAATCGAGAGAATCAAGAAGGAGAATGACAATATGCAGATTGAGCTCAGGCACTTGAAGGGAGAAGATATTACATCTTTGCATCACAAAGACCTGATCGCTCTTGAAGATGCACTTGAGAATGGACTTTTGAACATTCGCAGCAAAAAGTCCGAGATCCCAAAGATGATGAGCAAACATGGACAAATATTAGAGGAGGAGAACAGGCACCTCCAGTATATATTGCAACAAACTGAGATGGCAGCTATGGAAGGGAATGTGGCAGATCAGATGGAC---AATGGGTACCATCAGAGAATGAGAGACTACAACCAACCAGATCAT---CAGGTGCCCTTTGCATTTCGCGTTCAGCCAATTCAGCCGAATCTACACGAGAGGATT

>Ilex aquifolium GQ141105

AACAGGCAGGTGACGTATTCGAAGCGGCGGAATGGGATCATGAAGAAGGCTAAGGAGATCACTGTTCTTTGTGATGCTCAAGTTTCTCTTGTAATCTTCGCTAGTTCTGGGAAGATGCACGAGTACTGTAGCCCTTCCACTACGCTAATTGAGATGTTGGATCGATACCACCATGCGTCAGGGAAAAAGTTGTGGGATGCTAAGCATGAGAATCTCAGCAATGAAATTGATAGAGTCAAGAAAGAGAACGACAGTATGCAGATTGAGCTCAGGCACTTGAAGGGGGAGGACATCCAATCTCTGCAATACAAAGAACTCATGGCTATAGAGGATGCCCTTGAAATCGGGCTTTCTGGTATTCGCAACAAGCAGATGGAGATCTTCAAGATGAAGACAAAAAATAAACAAATGCTAGAGGAGGAGAATCAGGAGCTCATTAGCATCTTGCACCAGCATGAGATGGCAGCTATGGATGGTAATGTGAGAGAG---GTCCAG---AGTGGGTATCATCAAACAGTGAGAGACTAC---CAATCT---------CAGATGCCTTTTGCCTTCCGGGTTCAGCCCATTCAACCAAATTTGCAAGACAGAATT

>Nyssa sylvatica 1 GQ141111

---CGGCAAGTGACGTTCTGCAAGCGGAGGAATGGGATCATGAAGAAGGCTAAGGAGATCACTGTTCTCTGCGATGCTCATGTCTCTCTTGTTATCTTCGCTAGTTCTGGCAAGATGCATGAGTACTGTAGCCCTTCTACTAACTTAATTGACATCTTGGATCGATACCACAAGCAGTCTGGGAAGAGGTTGTGGGATGCTAAACATGAGAATCTCAGCAATGAACTTGATAGAATCAAGAAAGAGAATGACAGCATGCAGATTGAGCTCAGGCACCTGAAGGGGGAAGATATCACATCTTTGCACTACAAAGAGCTCATGGCCATAGAAGAAGCACTAGAGAATGGACTTGCAGGTGTTCGCAACAAACAGATGGAAATCCACGACATGAAGACTAAAAATGATAAAATGCTGGAGGAAGAGAACAAGCACCTCAAGTACATTTTGCACCAACAAGAGATG---GCTTTGAATGGTAACGTGAGAGAC---ATGGAG---AATGGATATCATCAGAAAGTGAGAGACTTC---CAGCCC---------CAGATGCCTTTTGCCTTCCGTGTGCAGCCAATTCAACCAAATTTACAGGAGAGAATG

>Nyssa sylvatica 2 GQ141109

---CGGCAGGTGACGTTCTGCAAGCGGAGGAATGGGCTCATGAAGAAGGCTAAGGAGATCACTGTTCTCTGTGATGCTCATGTCTCTCTCGTTATCCTTGCTAGTTCTGGCAAGATGCACGAGTACTGTAGCCCTTCTACTAATTTGATTGACGTCTTGGATCGATACCAGAAGCAGTCTGGGAAGAAGTTGTGGGATGCTAAACATGAGAATCTCAGCAATGAAATTGATAGAATCAAGAAAGAGAATGACAGCATGCAGATTGAGCTCAGGCACCTGAAGGGGGAAGATATCACATCTTTGCACCACAAAGACCTCATGGCCATAGAGGAAGCACTTGAGAATGGGCTTGCGAGTGTTCGTGACAAACAGTTGGAAATCTACAAGATGATAAAGAAACATGATAAAATGCTGGAGGAGGAGAACAAGCACCTGAACTACAAGTTGCGCTGGCAGCAAGAGATGGCTATGGATGGTAATCTGAGGGAT---TTGGAG---AATGGATATCTTCAAAAAGTGAGAGACTTC---CAGCCC---------CAGATGCCTTTCGCCTTCCGCGTGCAGCCAATTCAGCCAAATTTACAGGAAAGAATT
