# Supplementary figures and images for: The double-corolla phenotype in the Hawaiian lobelioid genus Clermontia involves ectopic expression of PISTILLATA B-function MADS box gene homologs
Source: EvoDevo. 2012 Nov 1;3:26. doi: 10.1186/2041-9139-3-26 (PMC3564722; doi:10.1186/2041-9139-3-26)

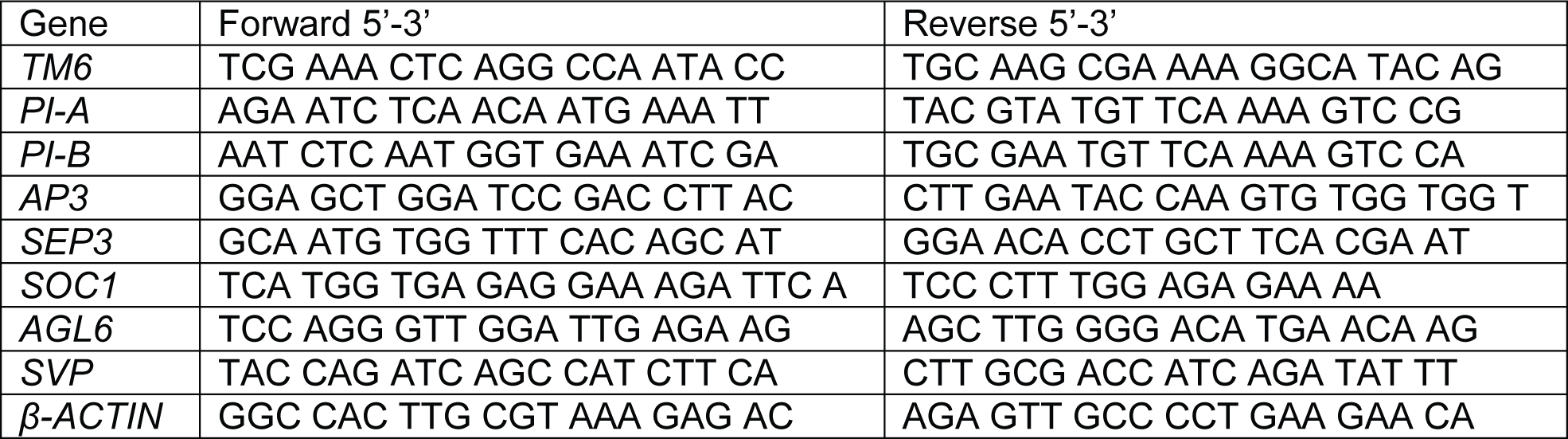

Supplement: Additional file 3 — Primers used for quantitative RT-PCR. Amplification of target and control genes was performed using the primer sequences listed in table format. [file 2041-9139-3-26-S3.png]

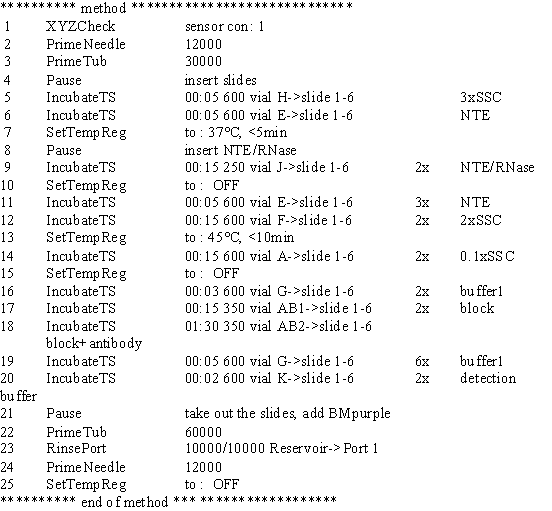

Supplement: Additional file 4 — Command file for in situ hybridization. The method for in situ hybridization using the InsituPro Vsi 3.0 (Intavis AG) liquid handling robot is presented. [file 2041-9139-3-26-S4.png]

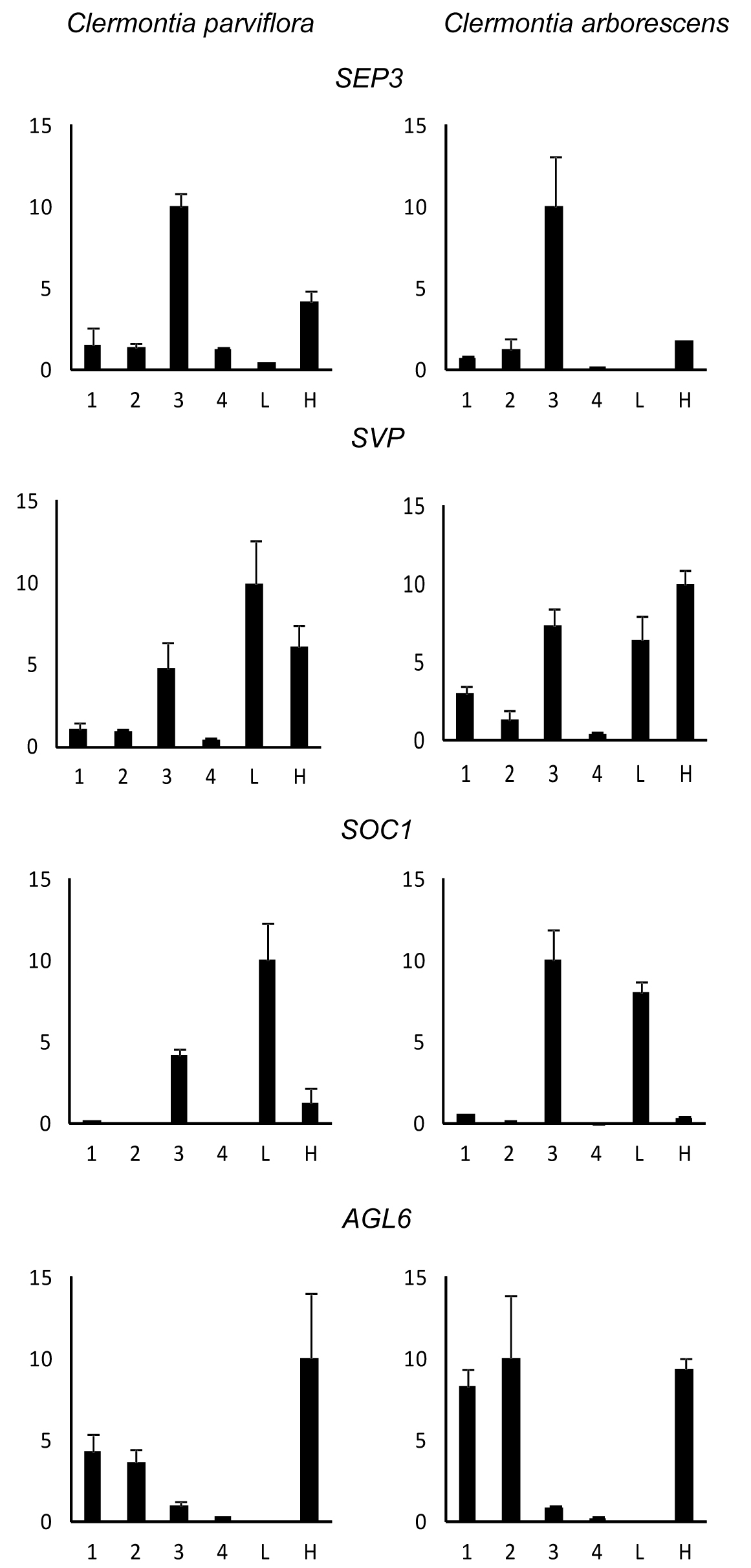

Supplement: Additional file 7 — Quantitative RT-PCR for MADS-box containing genes. Expression patterns of SVP, SOC1, AGL6, and SEP3 homologs in floral whorls 1,2,3,4, leaf and hypanthium for sepal-petal C. arborescens and double-corolla C. parviflora show no significant differences between species. Expression levels are shown as fold differences relative to β-actin and represent the mean and standard deviation of three independent experiments with two replicates each. [file 2041-9139-3-26-S7.jpeg]

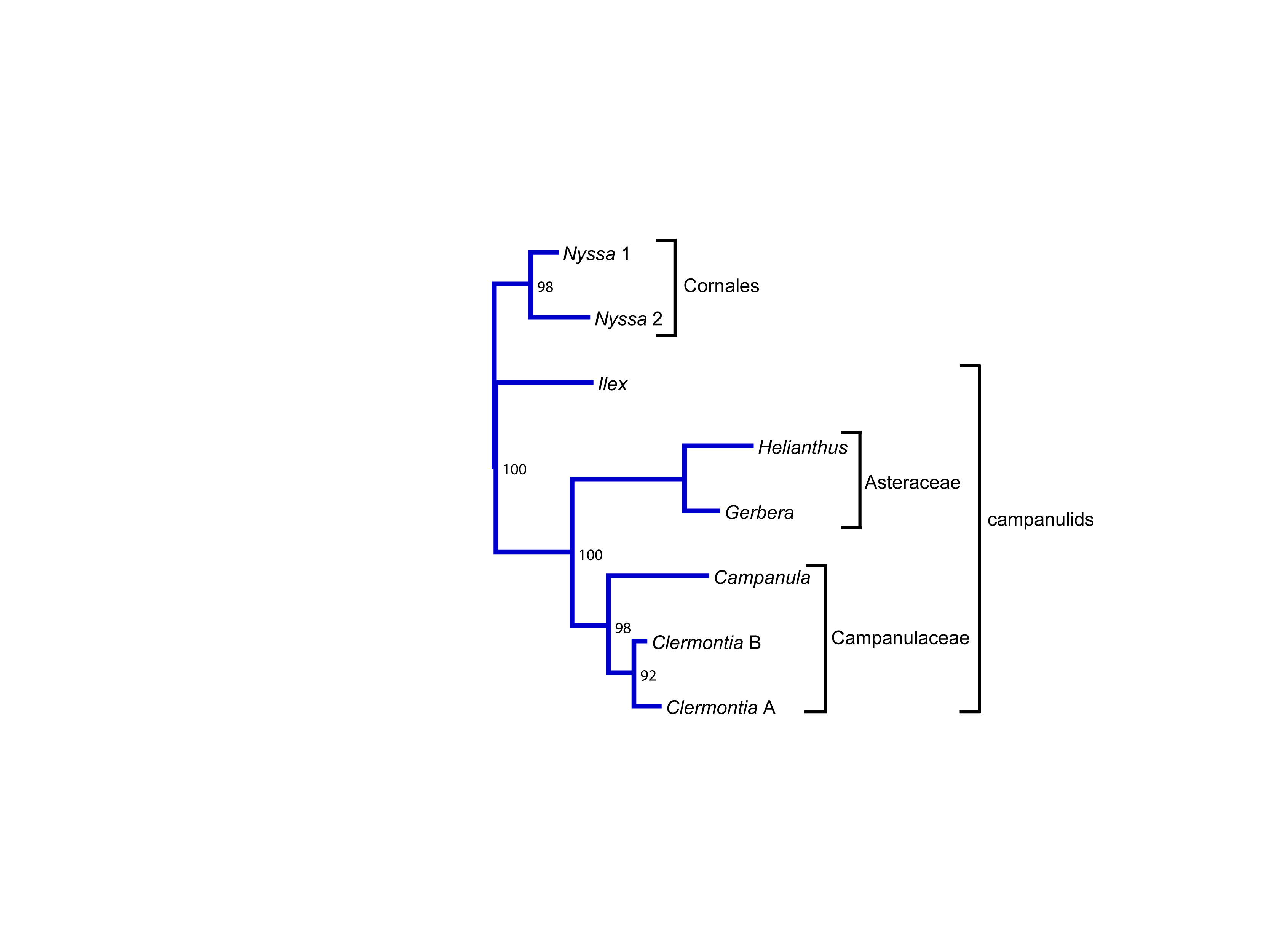

Supplement: Additional file 8 — Phylogenetic analysis of PISTILLATA-like coding sequences from Clermontia and outgroups. Based on these data the two Clermontia duplicates clearly arose after common ancestry with Campanula, a close sister taxon in Campanulaceae, although it cannot be certain that additional copies remain undetected in this and other species, such as with Nyssa in Cornales. No other PI-like sequences from the same plant individuals represented here are available in GenBank. Clermontia parviflora PI-like copies are shown. The single tree of maximum likelihood is figured with bootstrap values indicated. [file 2041-9139-3-26-S8.png]
